# MiR-125b regulates inflammation in bovine mammary epithelial cells by targeting the NKIRAS2 gene

**DOI:** 10.1186/s13567-021-00992-0

**Published:** 2021-09-17

**Authors:** Zhuo-Ma Luoreng, Da-Wei Wei, Xing-Ping Wang

**Affiliations:** School of Agriculture, Ningxia University, Yinchuan, 750021 China

**Keywords:** miR-125b, bovine mammary epithelial cell, inflammation, NF-κB

## Abstract

Mastitis is a complex inflammatory disease caused by pathogenic infection of mammary tissue in dairy cows. The molecular mechanism behind its occurrence, development, and regulation consists of a multi-gene network including microRNA (miRNA). Until now, there is no report on the role of miR-125b in regulating mastitis in dairy cows. This study found that miR-125b expression is significantly decreased in lipopolysaccharide (LPS)-induced MAC-T bovine mammary epithelial cells. Also, its expression is negatively correlated with the expression of NF-κB inhibitor interacting Ras-like 2 (NKIRAS2) gene. MiR-125b target genes were identified using a double luciferase reporter gene assay, which showed that miR-125b can bind to the 3′ untranslated region (3′ UTR) of the NKIRAS2, but not the 3′UTR of the TNF-α induced protein 3 (TNFAIP3). In addition, miR-125b overexpression and silencing were used to investigate the role of miR-125b on inflammation in LPS-induced MAC-T. The results demonstrate that a reduction in miR-125b expression in LPS-induced MAC-T cells increases NKIRAS2 expression, which then reduces NF-κB activity, leading to low expression of the inflammatory factors IL-6 and TNF-α. Ultimately, this reduces the inflammatory response in MAC-T cells. These results indicate that miR-125b is a pro-inflammatory regulator and that its silencing can alleviate bovine mastitis. These findings lay a foundation for elucidating the molecular regulation mechanism of cow mastitis.

**Supplementary Information:**

The online version contains supplementary material available at 10.1186/s13567-021-00992-0.

## Introduction

Bovine mastitis is an inflammatory disease caused by pathogenic infection of mammary tissue and is one of the most common diseases found on dairy farms [1]. Infection of the mammary tissue by pathogenic microorganisms increases the secretion of inflammatory cytokines by bovine mammary epithelial cells (bMECs). This induces cellular immune and inflammatory responses, leading to the recruitment of white blood cells to the infection site [2–4]. Ultimately, a potent immune system is activated, preventing the bacteria from further invading the mammary tissue and eliminating existing infections to restore the normal function of the mammary glands [4]. These findings show that the molecular mechanism behind the occurrence and development of mastitis is very complex. In recent years, researchers have screened mastitis related genes and studied their molecular regulation [5, 6]. However, most of the currently identified genes cannot be effectively used in molecular breeding for mastitis resistance. Therefore, it is imperative to identify additional genes and explore the molecular mechanism behind their link to mastitis.

MicroRNA (miRNA) is a recently discovered class of endogenous non-coding RNA with an approximate length of 18–22 nt. MiRNA enables rapid cellular responses upon cell stimulation by extracellular signals and consequently fine-tune the regulation of many biological processes including inflammation [7–10]. As an important member of the miRNA family, miR-125b regulates the expression of TNF-α [11, 12], TNFAIP3 [13, 14], and NKIRAS2 [15] in humans. These three genes are related to the cellular inflammatory response. Nevertheless, there is less similarity between target genes of miR-125b in bovines and humans, suggesting that the function of human miR-125b may not be completely consistent with that of cattle. To date, there are no reports on the regulation of inflammation or mastitis by miR-125b in dairy cows. However, miR-125b is significantly down-regulated in mammary tissue obtained from dairy cows clinically diagnosed with mastitis [16] or in *Staphylococcal* enterotoxin B (SEB) treated bMECs [17], suggesting that bovine miR-125b may play a critical role in mastitis. To explore the regulatory role of miR-125b in bovine mastitis, we conducted a study on the molecular mechanism of miR-125b at the cellular level. This study lays a foundation for future studies on the molecular network behind bovine mastitis.

## Materials and methods

### Cell culture

Bovine mammary epithelial cells line MAC-T [18] was used to examine the expression level of miR-125b related genes and study the molecular regulatory mechanism of miR-125b on cell inflammation. The cells were cultured in DMEM/F12 medium (GE Healthcare Life Sciences Hyclone Laboratories, South Logan, Utah) containing 10% FBS (BI, Israel Beit Haemek Ltd.). Human embryonic kidney (HEK) 293 T cells that were used to identify bovine miR-125b target genes were cultured on a high-glucose DMEM (GE Healthcare Life Sciences Hyclone Laboratories, South Logan, Utah) containing 10% FBS (BI, Israel Beit Haemek Ltd.). The two types of cells were cultured in vitro in a 5% CO_2_ incubator at 37 °C.

### Induction of inflammation in MAC-T Cells

Inoculated MAC-T cells were cultured in vitro in a 6-well cell culture plate. When the cell monolayer grew to 60–70% confluence, the cell culture medium was changed and 50 ng/μL LPS was added to each well (liquid medium culture served as a negative control) [19]. The cells were further incubated for 3 h to allow the induced MAC-T to produce an inflammatory response.

### Target gene prediction and identification

The online tool TargetScan 7.0 was used to predict the target genes of bovine miR-125b. Dual-luciferase reporter assay was performed to identify the target gene in 293 T cells as described in our previous study [20]. In brief, 3′ UTR and its site-specific mutated forms of the predicted miR-125b target mRNA were amplified using gene -specific primers (Table 1). The PCR amplicons were purified then cloned into the Xho I-Not I site of the psiCHECK™-2 vector (Promega Corp., Madison, USA), containing renilla and firefly luciferase reporter genes. The recombinant plasmid constructs (CHECK2-3′UTR-*wt* and CHECK2-3′UTR-*mut*) were extracted using a Endo-free Plasmid Mini Kit (Omega Bio-Tek, Inc., USA) and verified by DNA sequencing.

**Table 1:** **Primers used for amplification of NKIRAS2 and TNFAIP3 for psiCHECK™-2 recombinant vector construction**.

Primer name	Primer sequence (5′-3′)	Size (bp)
NKIRAS2-*wt*	F: 5′ TTCCGCTCGAGTGGGGTAGTTGTGGGTCATT 3′ R: 5′ AATACTAAGCGGCCGCCATTTCATTCCACCTCACCC 3′	791
NKIRAS2-*mut*	F: 5′ TGTAACTCTTCCCCTAGCTTCCTTTTCCTGCT**T**AG 3′ R: 5′ TGACATGCTCCCT**A**AGCAGGAAAAGGAAGC 3′	–
TNFAIP3-*wt*	F: 5′ CTTGTGCCCTCGAGAAACCCAGAGCCAGTCCC 3′ R: 5′ TTATCATTGCGGCCGCGCTCAGCCTCAGCATCAC 3′	270

The underlined sequence is the restriction endonuclease site for recombinant vector construction. The letter in bold indicates the introduced mutation

Then, 0.5 μg CHECK2-3′UTR-*wt* + 0.5 μg of miR-125b mimic, 0.5 μg of CHECK2-3′UTR-*wt* + 0.5 μg of NC_ mimic (Cy3 labeled, negative control group), and 0.5 μg of CHECK2_3′UTR (blank control group) were separately transfected into 293 T cells using 2.0 μL of X-tremeGENE HP DNA Transfection Reagent (Roche, Penzberg, Germany) according to the manufacturer’s protocol. Transfection efficiency was assessed by fluorescence microscopy (Additional file 1: A, B). Renilla luciferase activity and firefly luciferase activity were examined at 48 h post-transfection using the Dual-Luciferase Reporter Assay System (Promega Corp., Madison, USA), according to the manufacturer’s instructions. A significant reduction in the relative activity (Renilla/Firefly) of CHECK2-3′UTR-*wt* + miR-125b mimic group was used to determine whether the predicted target gene is indeed the target gene of miR-125b.

### Transfection of MAC-T cells

Bovine miR-125b mimic and inhibitor, and a Cy3-labeled negative control (NC, mismatched negative control mimic) were synthesized by Guangzhou RiboBio Co., Ltd. and dissolved in sterile RNase-free ddH_2_O. When inoculated MAC-T cells were cultured in 6-well plates until 50–60% confluence, the mimic, inhibitor, and negative control (NC) of bovine miR-125b were respectively transfected into MAC-T using the X-tremeGENE HP DNA Transfection Reagent (Roche, Penzberg, Germany) according to the manufacturer’s instructions. The final concentrations of inhibitor, mimic, and NC were 150 nM, 75 nM, and 75 nM, respectively. Cy3-labeled NC and qPCR of miR-125b were used to evaluate the efficiency of transfection.

### Total RNA extraction and reverse transcription

Total RNA was extracted from MAC-T cells using RNAiso Plus reagent (Takara Biomedical Technology (Beijing) Co., Ltd.) according to the manufacturer’s instructions. The RNA was eluted in sterile RNase-free ddH_2_O. The RNA concentration and purity were determined using a continuous wavelength microplate reader (Biotek Synergy SLXA, USA). The RNA sample concentration was adjusted to 1000 ng/μL. Reverse transcription (RT) of miR-125b and related mRNA was performed using PrimeScript ™ RT reagent Kit with gDNA Eraser (Takara Biomedical Technology (Beijing) Co., Ltd.). For RT reaction system and conditions, refer to Additional file 2.

### Quantitative PCR

The expression of miR-125b, NKIRAS2, nuclear factor kappa-B (NF-κB), Interleukin- 6 (IL-6), and tumor necrosis factor α (TNF-α) in MAC-T cells was measured using stem-loop quantitative PCR (qPCR) and standard qPCR. All the qPCR experiments were performed using TB Green® Premix Ex Taq ™ II kit (Tli RNaseH Plus) (Takara Biomedical Technology (Beijing) Co., Ltd.) on a Bio-Rad CFX96 Touch real-time PCR instrument, as per the MIQE guidelines [21]. The qPCR results were normalized to glyceraldehyde-3-phosphate dehydrogenase (GADPH) and ubiquitously expressed prefoldin like chaperone (UXT) gene expressions (internal control) [22]. The relative expression was calculated using the 2^−△△ct^ method [23]. The primers used for qPCR are listed in Table 2. The qPCR reaction system and conditions are described in Additional File 2.

**Table 2:** **Primer sequences for RT-qPCR**.

Gene symbol	Primer sequence	Amplicon (bp)	References
NKIRAS2	F: 5′-GGAGCCCTTCGTCTACCT-3′ R: 5′-GCACCACTTCAGCCATCC-3′	111	–
NF-κB	F: 5′-AAGAGCCCTTTCAATGGACC-3′ R: 5′-GTGCTGAGAGATGGCGTAAA-3′	128	–
IL-6	F: 5′-CTGGGTTCAATCAGGCGAT-3′ R: 5′-CAGCAGGTCAGTGTTTGTGG-3'	205	[19]
TNF-α	F: 5′-TCTGGGCAGGTCTACTTTG-3′ R: 5′-CCTGAGCCCATAATTCCCT-3′	139	–
UXT	F: 5′-CATTGAGCGACTCCAGGAAG-3′ R: 5′-GGCCACATAGATCCGTGAAG-3′	112	[22]
GADPH	F: 5′-GGCATCGTGGAGGGACTTATG-3′ R: 5′-GCCAGTGAGCTTCCCGTTGAG-3′	186	[22]
miR-125b	RT: 5′-GTCGTATCCAGTGCAGGGTCCGAGGTATTC GCACTGGATACGACTCACAAGT-3′ F: 5′-GCATCCTCCCTGAGACCCTA-3′ R: 5′-CAGTGCAGGGTCCGAGGTAT-3′	– 64	–

F (Forward) primer and R (Reverse) primer were used for qPCR assays. RT primer is used for reverse transcription of miR-125b.

### Western blot

Western blot was conducted to measure protein expression in MAC-T cells. Extraction of total cellular protein was performed as follows: cell lysis buffer containing 1% PMSF (Sangon Biotech Co., Ltd., Shanghai, China) was used to rupture cells, and Bicinchoninic Acid Protein Assay kit (Sangon Biotech Co., Ltd., Shanghai, China) was used to quantify total protein concentration according to the manufacturer’s instructions.

An equal amount of total protein (25 µg) from each sample was separated using 12% SDS-PAGE electrophoresis, transferred to a nitrocellulose membrane (Pall Corporation, New York, USA), blocked with 5% reconstituted skimmed milk powder, and incubated with primary antibody overnight at 4 °C. The next day, the membrane was washed three times, then incubated with HRP-labeled secondary antibody for 1 h. The membrane was again washed three times then developed using ECL substrates (Beyotime Biotech., Shanghai, China). A fully automated chemiluminescence imaging analysis system (Tanon 5200, Tanon Science & Technology Co., Ltd., Shanghai, China) was used for imaging. Image J software was used to analyze Western blot images.

All Western blot primary and secondary antibodies, including IL-6 (1: 500, cat. no. DF6087), phospho-NF-κB p65 (1: 500, cat.no. AF2006), TNF-α (1: 500, cat. no. AF7014), and β-actin (1: 500, cat. no. AF7018) were purchased from Affinity Bioscience (OH, USA). Goat anti-mouse IgG-HRP (1: 5000, cat. no. sc-2005) and goat anti-rabbit IgG-HRP (1: 5000, cat. no. sc-2030) were purchased from Santa Cruz Biotechnology, Inc. (Dallas, TX, USA). Beta-actin was used as an internal reference for all Western blots.

### Statistical analysis

Gene and protein expression levels were evaluated in triplicates. All gene and protein expression data are expressed as mean ± SD. GraphPad Prism 8.0 software (GraphPad Software, Inc., La Jolla, CA, USA) was used for statistical analysis and ordinary one-way ANOVA multiple comparisons were used to test the significance of differences. If the corrected *p*-value was less than 0.05, the difference was deemed significant. If the corrected *p*-value was less than 0.01, then the difference was deemed highly significant.

## Results

### Differential expression of miR-125b and NKIRAS2 in LPS-induced MAC-T cells

**Figure 1:**
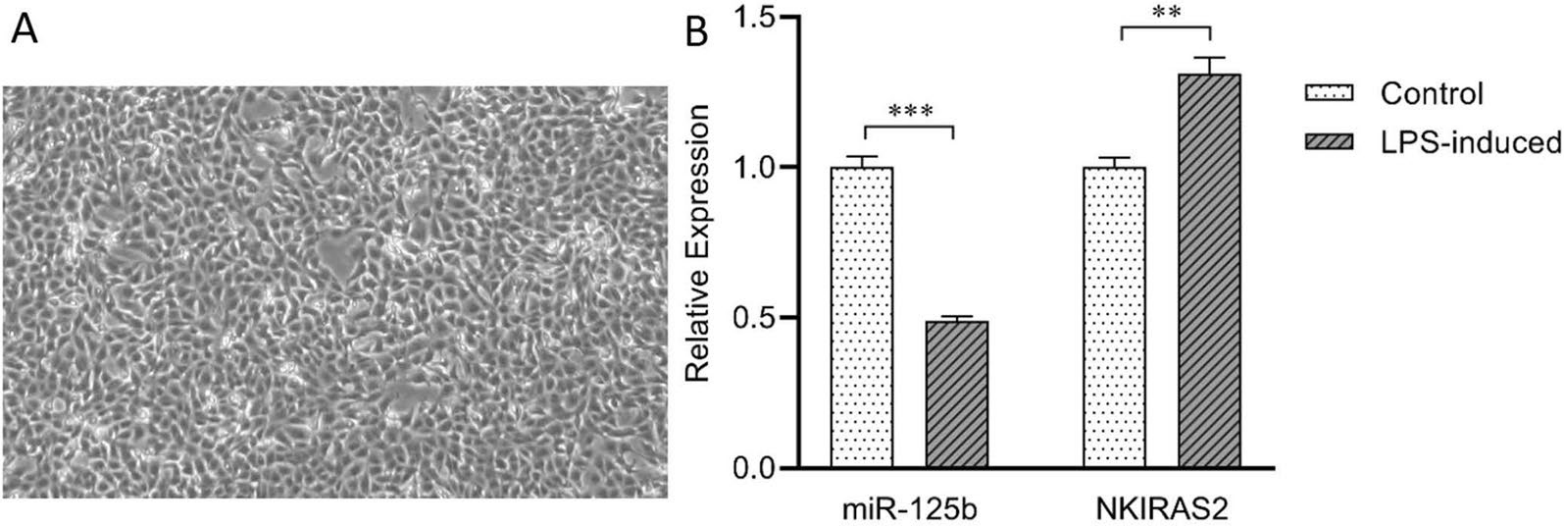
**Expression of miR-125b and *NKIRAS2*in LPS-induced MAC-T cells.A** LPS-induced-MAC-T cells (40 ×). **B** MAC-T cells were challenged with LPS (50 ng/μL) for 3 h, then qPCR was used to measure the expression level of miR-125b and NKIRAS2. Data are Mean ± SD. **p value < 0.01, ***p value < 0.001.

Aiming at evaluating the variation in expression of miR-125b and of NKIRAS2 upon LPS induction, LPS-induced MAC-T cells were collected and total RNA was extracted to detect the expression of miR-125b and NKIRAS2 by qPCR. Compared with the control group, the expression level of miR-125b in LPS-induced MAC-T cells was extremely significantly down-regulated (*p* < 0.001), while NKIRAS2 expression was extremely significantly up-regulated (*p* < 0.01) (Figure 1). These results show that upon induction of an inflammatory response in MAC-T cells, crosstalk between miR-125b and NKIRAS2 expression occurs. This explains the relationship between miR-125b and inflammatory response or cellular immune response, considering that NKIRAS2 inhibits NF-κB. Combined with the targeted regulatory relationship between human miR-125b and NKIRAS2 [14], we speculate that NKIRAS2 may be a target gene of miR-125b in dairy cows.

### NKIRAS2 is a target gene of miR-125b

**Figure 2:**
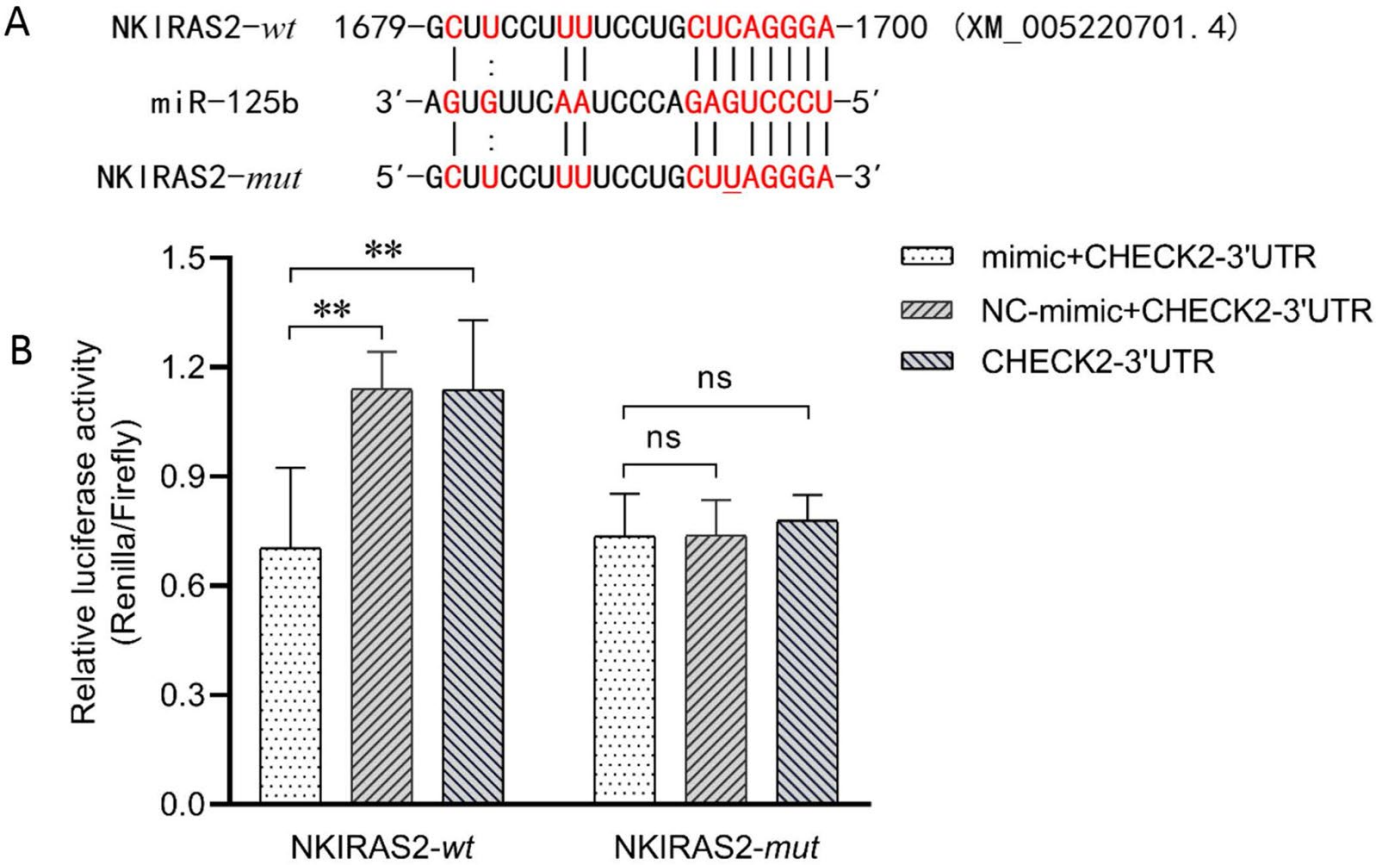
**Bovine miR-125b directly targets NF-κB inhibitor interacting RAS-like 2 (NKIRAS2) gene by binding to its 3′ untranslated region (3′ UTR).A** The predicted miR-125b binding sites within 3′ UTR of NKIRAS2 and the experimentally introduced mutations are shown. **B** Human embryonic kidney (HEK) 293 cells were transiently co-transfected with CHECK2-3′UTR dual-luciferase reporter recombinant vector, and bovine miR-125b mimic or NC_mimic. The data in legend are the relative luciferase activity (Renilla / Firefly), in which, renilla luciferase activity was normalized to the activity of firefly luciferase in the same recombination vector. Data are Mean ± SD. **: adjusted* p* value < 0.01; ns: adjusted *p* value > 0.05. NC_mimic = negative control, compared with miR-125b mimics; CHECK2_3′UTR = NKIRAS2-3UTR-wt (or -*mut*) and psiCHECK™-2 dual luciferase reporter recombinant vector.

To further explore the function of miR-125b, we used bioinformatics tools to predict the target gene of miR-125b. Our results show that miR-125b potentially binds to the 3′UTR of the NKIRAS2 gene via complementary bases (Figure 2A). Dual-luciferase reporter assay was performed in 293 T cells to determine whether or not NKIRAS2 is a target gene of miR-125b. The results show that compared with the blank control and negative control, the relative luciferase activity in the CHECK2-3′UTR-*wt* + mimic group was significantly reduced (*p* < 0.01), while the NC_mimic + CHECK2-3′UTR-*wt* and CHECK2-3′UTR groups showed no significant difference in relative luciferase activity (*p* > 0.05) (Figure 2B). In addition, the CHECK2-3′UTR-mut vector was also co-transfected with the same miR-125b mimic, and the results of relative luciferase activity measurement showed no significant difference among the groups (*p* > 0.05) (Figure 2B). Overall, these results indicate that miR-125b can directly bind the 3′ UTR of the NKIRAS2 gene at 1679–1700 bp (GenBank ID: XM_005220701.4) and inhibit luciferase activity.

### TNFAIP3 is not a target gene of miR-125b

**Figure 3:**
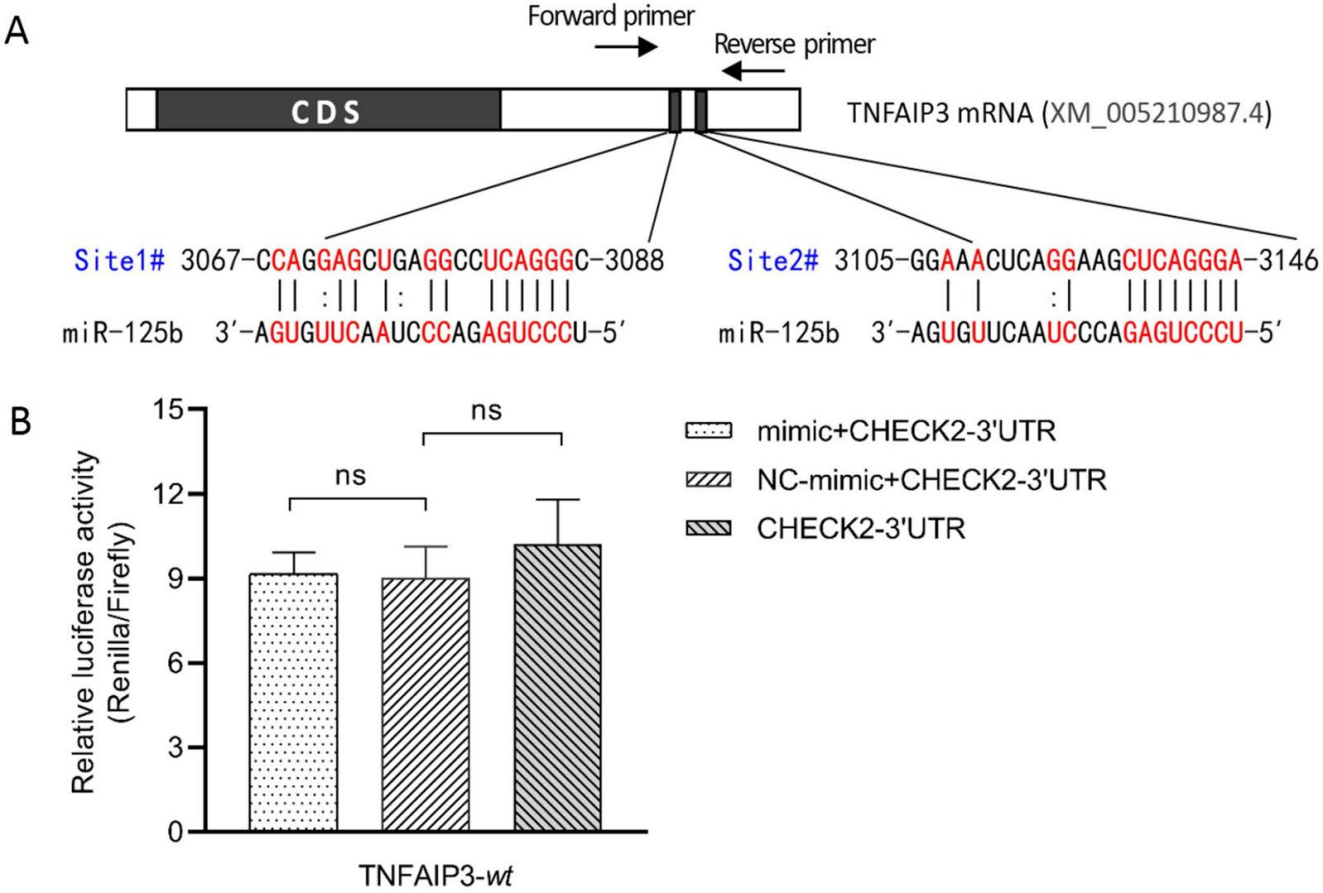
**The tumor necrosis factor alpha-induced protein 3 (TNFAIP3) gene is not directly targeted by bovine miR-125b.A** The sequence alignments of miR-125b and its putative targets in the 3′ untranslated region (UTR) of TNFAIP3, which have been identified as targets of miR-125b in human (has-miR-125b) [8, 12]. **B** Human embryonic kidney (HEK) 293 T cells were transiently co-transfected with CHECK2-3′ UTR dual-luciferase reporter recombinant vector, and bovine miR-125b mimic or NC_mimic. The data in legend are the relative luciferase activity (Renilla / Firefly), in which, renilla luciferase activity was normalized to the activity of firefly luciferase in the same recombination vector. Data are Mean ± SD. ns: adjusted *p* value > 0.05, as determined by one-way ANOVA multiple comparisons. NC_mimic = negative control, compared with miR-125b mimics; CHECK2-3′UTR = TNFAIP3-3UTR-*wt* and psiCHECK™-2 dual-luciferase reporter recombinant vector.

Previous studies have shown that human miR-125b can target the TNFAIP3 (also known as A20) [13, 14]. Interestingly, in our target gene prediction, we found two potential binding sites for miR-125b at the 3′ UTR of the TNFAIP3 (Figure 3A). One of the sites (Site # 2) is similar to the binding site found in humans. By sequence comparison, we discovered that while the sequences of mature miR-125b in humans and cows are completely identical (Additional file 3A), the homology of the TNFAIP3 gene between the two species is 81.79% (Additional file 3B) and the homology of the 3′UTR of the TNFAIP3 gene is only 69.45% (Additional file 4). As a result of the low degree of homology, results from human research may not be fully applicable to cows. Therefore, we conducted a dual luciferase reporter gene experiment to verify the two predicted binding sites using the same experimental protocol as that used for NKIRAS2 gene target verification in this study. We found no significant difference in relative luciferase activity between cells transfected with CHECK2-3′UTR-*wt *+ mimic, NC_mimic + CHECK2-3′UTR-*wt*, and CHECK2-3′UTR group (*p* > 0.05) (Figure 3B), indicating that TNFAIP3 is not a target of bovine miR-125b. This difference between human and cow may be because of base mutations in the 3′ UTR of the bovine TNFAIP3 gene, which reduces the efficiency of miR-125b binding.

### MiR-125b targets and inhibits NKIRAS2 gene expression in MAC-T cells

**Figure 4:**
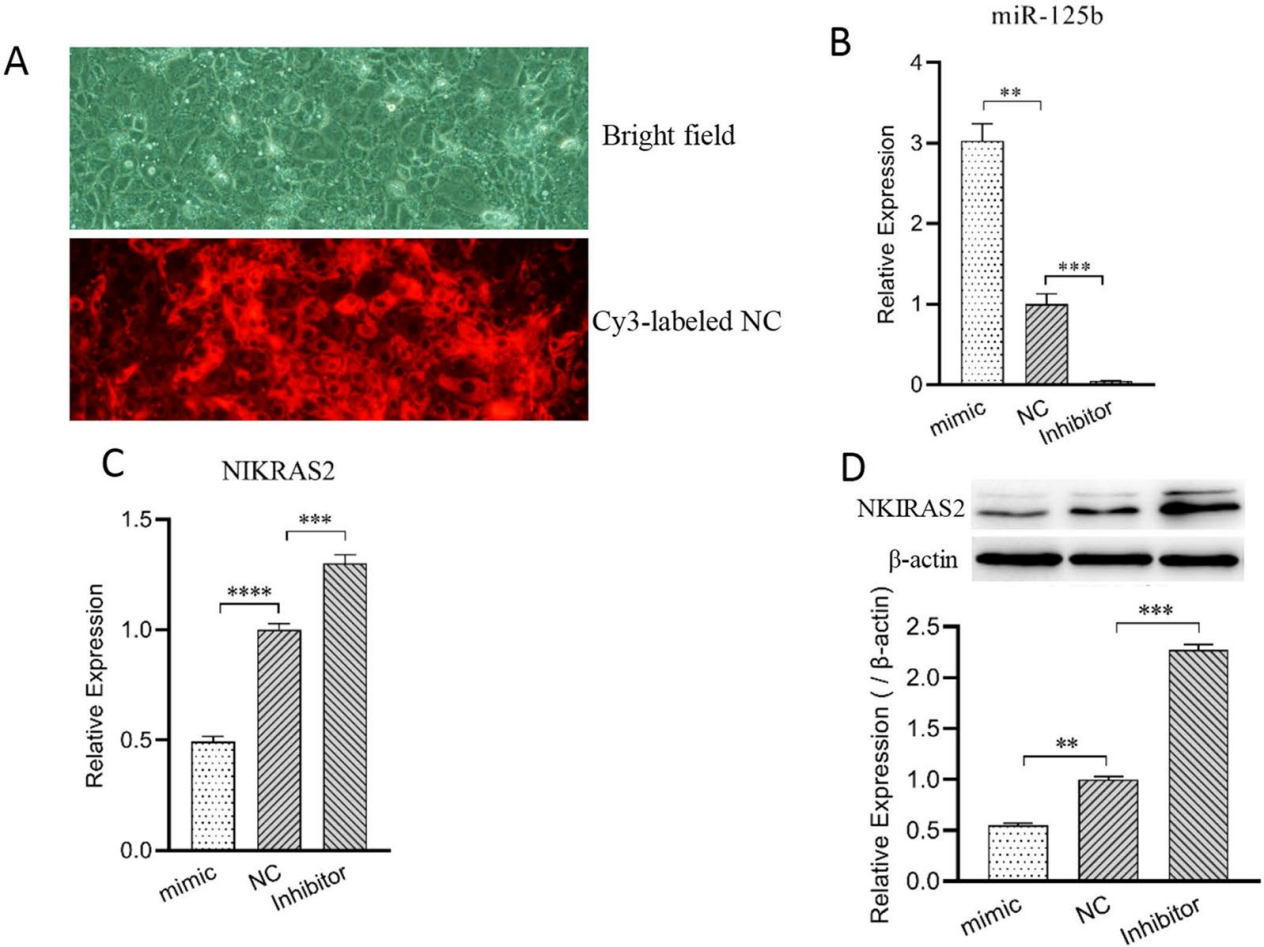
**Bovine miR-125b suppresses NKIRAS2 expression in MAC-T cells.A** Transfection efficiency of miR-125b mimic or inhibitor in MAC-T cells as observed by fluorescence microscopy. **B** The transfection efficiency of bovine miR-125b mimic or inhibitor in MAC-T cells as measured by qPCR. **C** qPCR analysis of NKIRAS2 in MAC-T cells transfected with miR-125b mimic or inhibitor. **D** Western blot analysis of NKIRAS2 in MAC-T cells transfected with miR-125b mimic or inhibitor. Western blot images were analyzed using the Image J software, and densitometry quantification of western blots were normalized to β-actin. Data are presented as mean ± SD. **: adjusted *p* value < 0.01, ***: adjusted* p* value < 0.001, ****: adjusted* p* value < 0.0001, as determined by as determined as determined by one-way ANOVA multiple comparisons. mimic = miR-125b mimic, Inhibitor = miR-125b inhibitor, NC = negative control, compared with miR-125b mimic and inhibitor.

To confirm whether miR-125b targets and regulates NKIRAS2 expression in MAC-T cells, we respectively transfected the miR-125b mimic, inhibitor, and NC mimic into MAC-T cells. The cells were then cultured in vitro for 48 h (Figure 4A) to ensure that miR-125b was either over-expressed or inhibited in the transfected cells compared to NC group (Figure 4B). Next, the mRNA and protein expression levels of the NKIRAS2 gene in MAC-T were measured using qPCR and Western blot. Compared with the NC group, the expression levels of the NKIRAS2 gene (Figure 4C) and protein (Figure 4D) in the mimic group were significantly down-regulated (*p* < 0.0001), while NKIRAS2 gene expression in the inhibitor group was significantly up-regulated (*p* < 0.001). These results indicate that miR-125b inhibits NKIRAS2 gene expression in MAC-T cells. Cognizant of these findings and the negative regulatory relationship between NKIRAS2 and NF-κB, we speculate that miR-125b may regulate the activity of NF-κB, which may be related to the regulation of MAC-T inflammation.

### MiR-125b promotes the expression of IL-6 and TNF-α genes in LPS-induced MAC-T by targeting the NKIRAS2 gene

**Figure 5:**
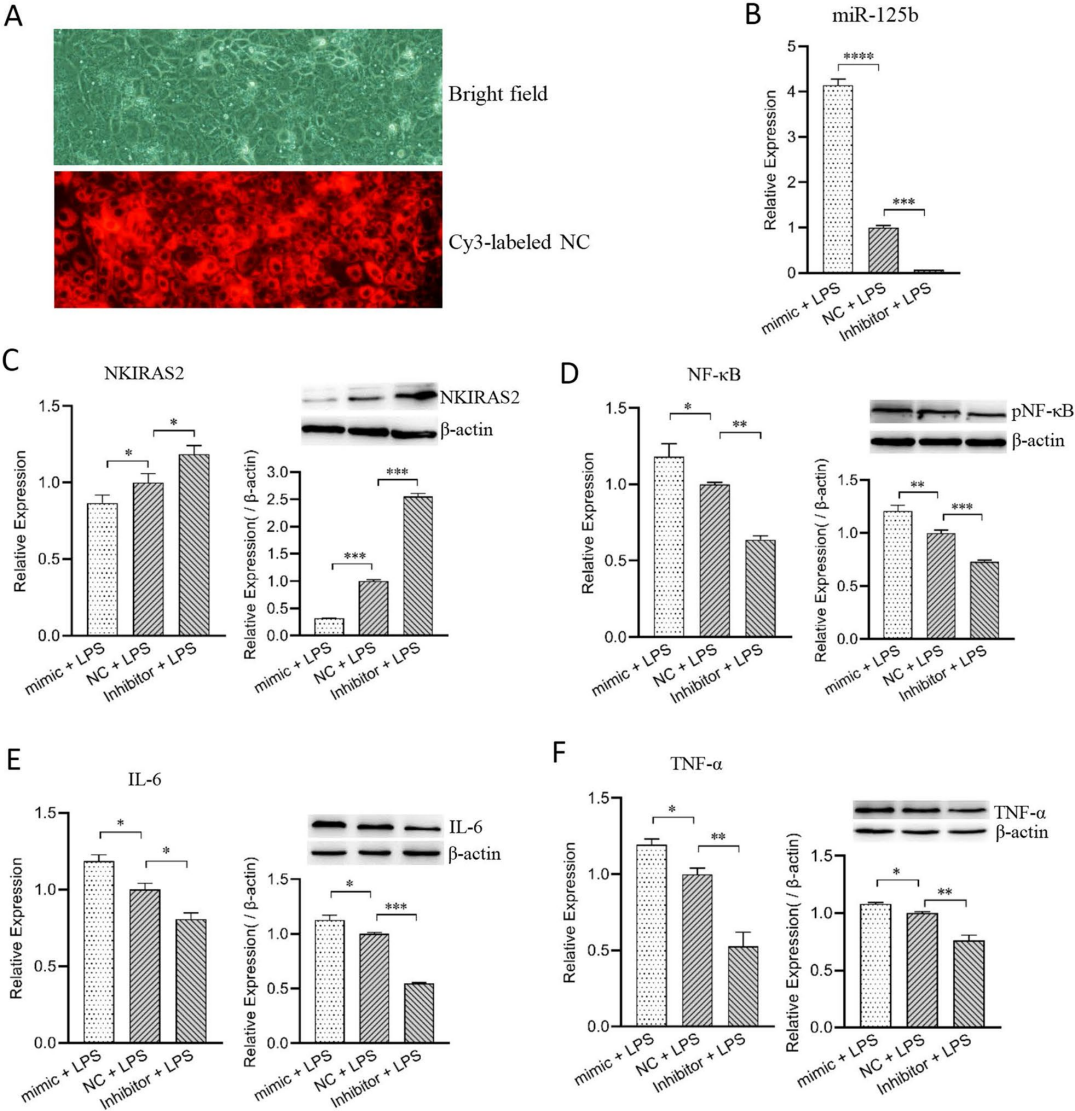
**Bovine miR-125b regulates the expression of NF-κB, TNF-α and IL-6 in LPS-induced MAC-T cells.** The MAC-T cells were transfected with miR-125b mimic, inhibitor or scrambled control (NC, Cy3-labeled). The transfection efficiency of bovine miR-125b mimic or inhibitor in MAC-T cells as measured by fluorescence micrograph (**A)** and qPCR (**B**). At 48 h post-transfection, MAC-T cells were challenged with LPS for 3 h. Cells were then collected to measure mRNA expression and production of NKIRAS2 (**C**), NF-κB (**D**), IL-6 (**E**), and TNF-α (**F**). Expression levels of mRNA and protein were determined by qPCR and Western blot, respectively. *p < 0.05, **p < 0.01, ***p < 0.001.

To study the role of miR-125b in the inflammatory response of MAC-T cells, we separately transfected miR-125b mimic and inhibitor into MAC-T cells (Figure 5A). This increased or silenced the expression of miR-125b in the cells 24 h later (Figure 5B). The cells were then treated with 50 ng/μL LPS for 3 h to induce an inflammatory response. Subsequently, the expression of inflammation-related genes in the cells was evaluated using qPCR and Western blot. In the mimic group, NKIRAS2 gene and protein expressions were significantly reduced compared with the NC group (*p* < 0.05) (Figure 5C). In contrast, the mRNA and protein expression of NF-κB, IL-6 and TNF-α were significantly increased (*p* < 0.05) (Figures 5D–F). Furthermore, the results of the inhibitor transfected group contradicted those observed in the mimic group. Collectively, these results indicate that miR-125b can inhibit the expression of the NKIRAS2 gene in MAC-T cells and promote the expression and activity of phosphorylated NF-κB (pNF-κB), thereby up-regulating the expression of intracellular inflammatory factors IL-6 and TNF-α.

## Discussion

The change in miRNA expression is part of the cell early response to stimulation by extracellular signals. This rapid response by miRNAs can regulate signal transduction pathways related to immune response and inflammatory response, thereby changing the level of expression of inflammatory factors and fine-tuning immune response and disease development [24–26]. LPS is a major component of *Escherichia coli* bacterial outer membrane and can induce inflammatory responses in certain cells. This study found that miR-125b expression is significantly reduced in LPS-stimulated MAC-T cells (Figure 1), which is consistent with LPS-induced changes in Raw 264.7 and C57BL/6 cells [11]. In addition, miR-125b expression in mammary tissue from dairy cows with clinical mastitis [16] and in SEB-treated bMECs [17] is also significantly down-regulated, suggesting that miR-125b may play a role in bMECs inflammation or in bovine mastitis.

Several lines of evidence indicate that miRNAs regulate the expression of target genes through complementary binding to specific bases on the target mRNA. Even highly conserved miRNAs may have different regulatory actions on target genes in different organisms or even in different tissues of the same organism [27]. To determine the function of bovine miR-125b, we identified and validated target genes of bovine miR-125b. Our results show that bovine miR-125b can target the 3´ UTR of the NKIRAS2. These results are consistent with those observed in mice and in humans [14, 15]. Human miR-125b can regulate NKIRAS2 gene in periodontal ligament cells [15], glioblastomas [14], and MARC-145 cells [28]. In addition, studies have shown that miR-125b can target TNFAIP3 [13, 14] and TNF-α [11, 12, 26] gene expression. MiR-125b also has important regulatory functions in tumor cell apoptosis, migration, osteoblast differentiation, and mouse RAW264.7 activation. Chen et al. [29] observed a negative correlation between the expression of bovine miR-125b and TNFAIP3 through qPCR analysis, and predicted that miR-125b might target the 3′ UTR of TNFAIP3 using TargetScan software. In this study, dual-luciferase reporter assay confirmed that bovine miR-125b does not directly target the TNFAIP3 gene. This discrepancy may be due to the difference between human and bovine TNFAIP3 gene 3′ UTR sequences and the conservation between human and bovine miR-125b sequences, resulting in the loss of targeted binding capacity. In conclusion, miR-125b may indirectly control TNFAIP3 expression by regulating other target genes. However, further studies are needed to confirm this speculation.

**Figure 6:**
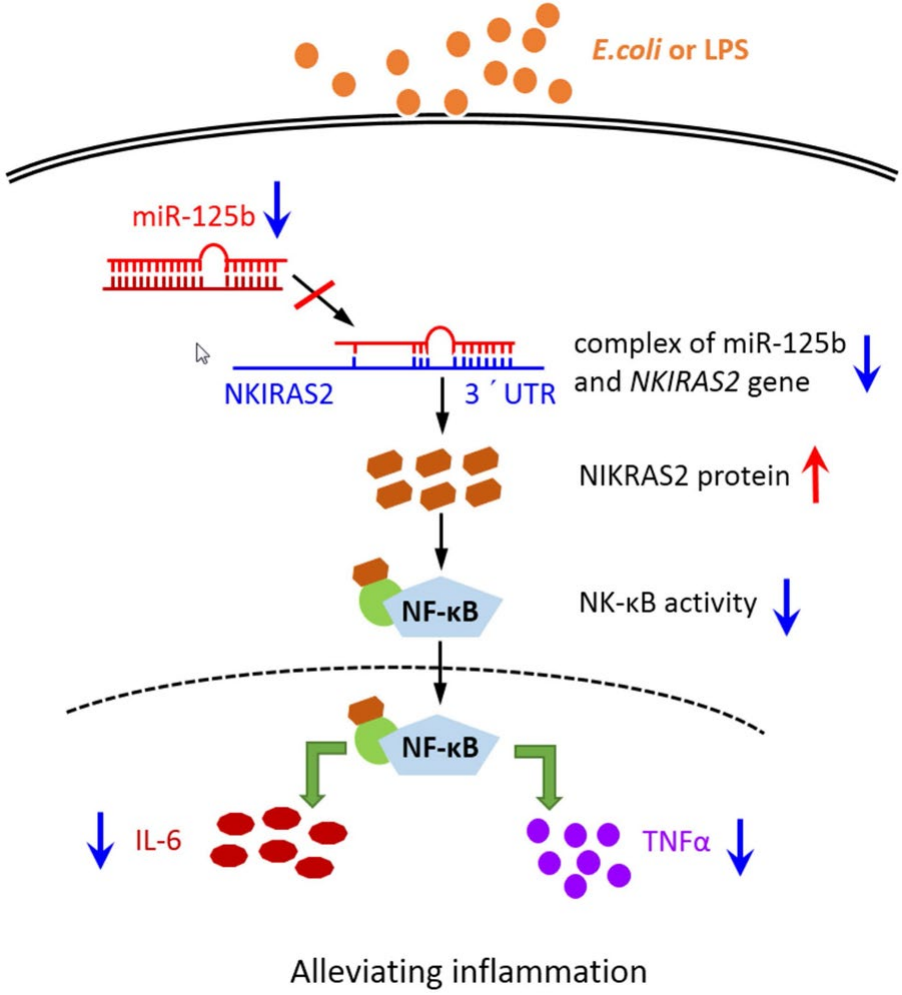
**Bovine miR-125b regulates inflammation of LPS-induced bovine mammary epithelial cells.** The down-regulation of miR-125b in LPS-induced bMECs increased the expression of NKIRAS2, eventually leading to a decrease in NF-κB activity and a decrease in the expression of IL-6 and TNF-α, alleviating cell inflammation.

Previous studies have shown that NKIRAS2 is a negative regulator of NF-κB [14, 30]. Additionally, LPS can activate the TLR4/NF-κB signaling pathway and promote innate immune and inflammatory responses [19]. Previous studies on the function of the miR-125b gene have mainly focused on its effects on tumor development and apoptosis, but there are very few reports on how it may regulate the inflammatory response. Our study found for the first time that bovine miR-125b targets the NKIRAS2 gene and inhibits its expression at the mRNA and protein levels. Furthermore, we tested the expression of inflammation related genes in LPS-induced MAC-T cells after miR-125b overexpression or silencing. The results indicate that miR-125b inhibits NKIRAS2 gene expression, consequently enhancing phosphorylated NF-κB activity, and promoting the expression and secretion of intracellular inflammatory factors IL-6 and TNF-α. Overall, our results demonstrate that miR-125b participates in regulating the inflammatory process in bovine mammary epithelial cells.

In summary, this study shows that bovine miR-125b expression is significantly down-regulated in LPS-induced MAC-T cells. This weakens its inhibitory effects on NKIRAS2 gene expression, and reduces the activity of NF-κB. Low NF-κB activity down-regulates the expression of the inflammatory factors IL-6 and TNF-α, consequently alleviating the overall inflammatory response in MAC-T cells (Figure 6). Collectively, our results indicate that miR-125b is a pro-inflammatory regulator and that its silencing can alleviate bovine mammary inflammation. This study provides a reference for the future development of molecular treatments against bovine mastitis.

## Supplementary Information

**Additional file 1:Transfection efficiency of bovine miR-125b mimic assessed by fluorescence microscopy.A** Bright field. **B** Cy3 labeled miR-125b NC_mimic in 293-T cells.

**Additional file 2:** **Protocol of total RNA isolation and RT-qPCR.**

**Additional file 3:Homology alignment of miR-125b and TNFAIP3 gene in bovine and human.A** Alignment of mature sequence of bovine miR-125b and human miR-125b. **B** Homology of complete TNFAIP3 mRNA in bovine and human.

**Additional file 4:** **Alignment of 3´ UTR of bovine TNFAIP3 and human TNFAIP3.**

## Abbreviations

NKIRAS2: NF-κB inhibitor interacting Ras-like 2
miRNA: microRNA
TNFAIP3: TNF-α induced protein 3
IL-6: interleukin- 6
TNF-α: tumor necrosis factor alpha
NF-κB: nuclear factor kappa-B
LPS: lipopolysaccharide
3′UTR: 3′Untranslated region of mRNA
SEB: *Staphylococcal* enterotoxin B
MAC-T: a cell line produced from primary bovine mammary alveolar cells by stable transfection with SV-40 large T-antigen
bMECs: bovine mammary epithelial cells
DMEM: Dulbecco's modified eagle medium
RT: reverse transcription
qPCR: quantitative real-time PCR
GADPH: glyceraldehyde-3-phosphate dehydrogenase
UXT: ubiquitously expressed prefoldin like chaperone
