# Correction to: MiR‑125b regulates inflammation in bovine mammary epithelial cells by targeting the NKIRAS2 gene

**DOI:** 10.1186/s13567-021-00999-7

**Published:** 2021-10-21

**Authors:** Zhuo‑Ma Luoreng, Da‑Wei Wei, Xing‑Ping Wang

**Affiliations:** School of Agriculture, Ningxia University, Yinchuan, 750021 China

## Correction to: Vet Res (2021) 52:122 https://doi.org/10.1186/s13567-021-00992-0

Following publication of the original article [1], the authors informed us that all references are sorted according to the author’s initials rather than the order in which they appear in the text, which makes the citation of references confused.

The original article has been corrected.
