# Supplementary figures and images for: MiR-125b regulates inflammation in bovine mammary epithelial cells by targeting the NKIRAS2 gene
Source: Vet Res. 2021 Sep 17;52:122. doi: 10.1186/s13567-021-00992-0 (PMC8447609; doi:10.1186/s13567-021-00992-0)

**
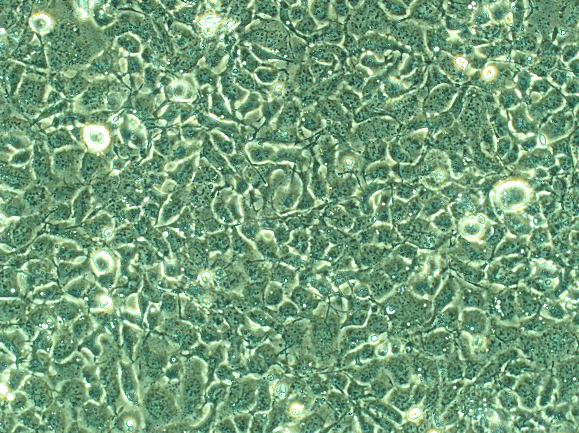

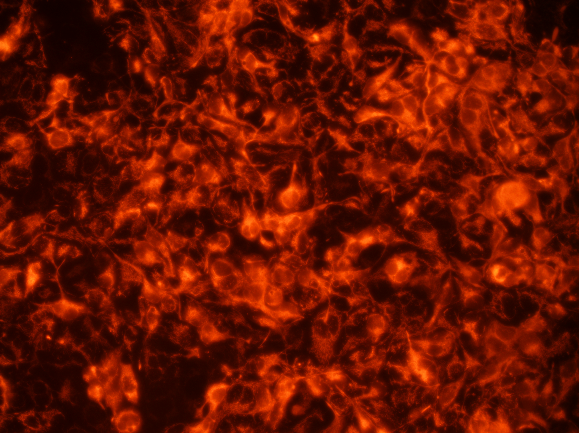
**

Cy3 labeled NC-mimic

Bright field

A

B

Supplement: Supplementary file 1 — Additional file 1:Transfection efficiency of bovine miR-125b mimic assessed by fluorescence microscopy.A Bright field. B Cy3 labeled miR-125b NC_mimic in 293-T cells. [file 13567_2021_992_MOESM1_ESM.docx]

A


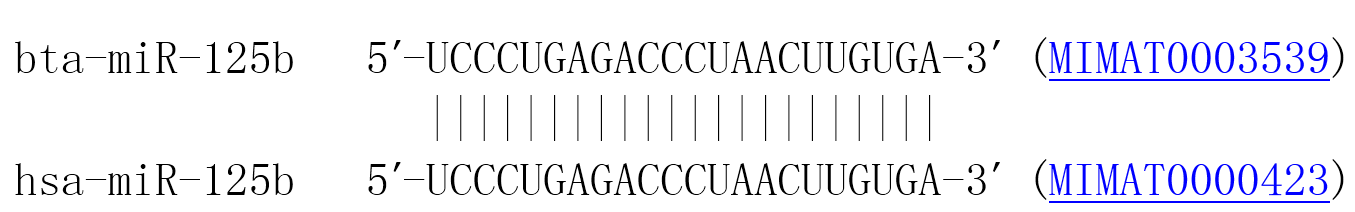


B


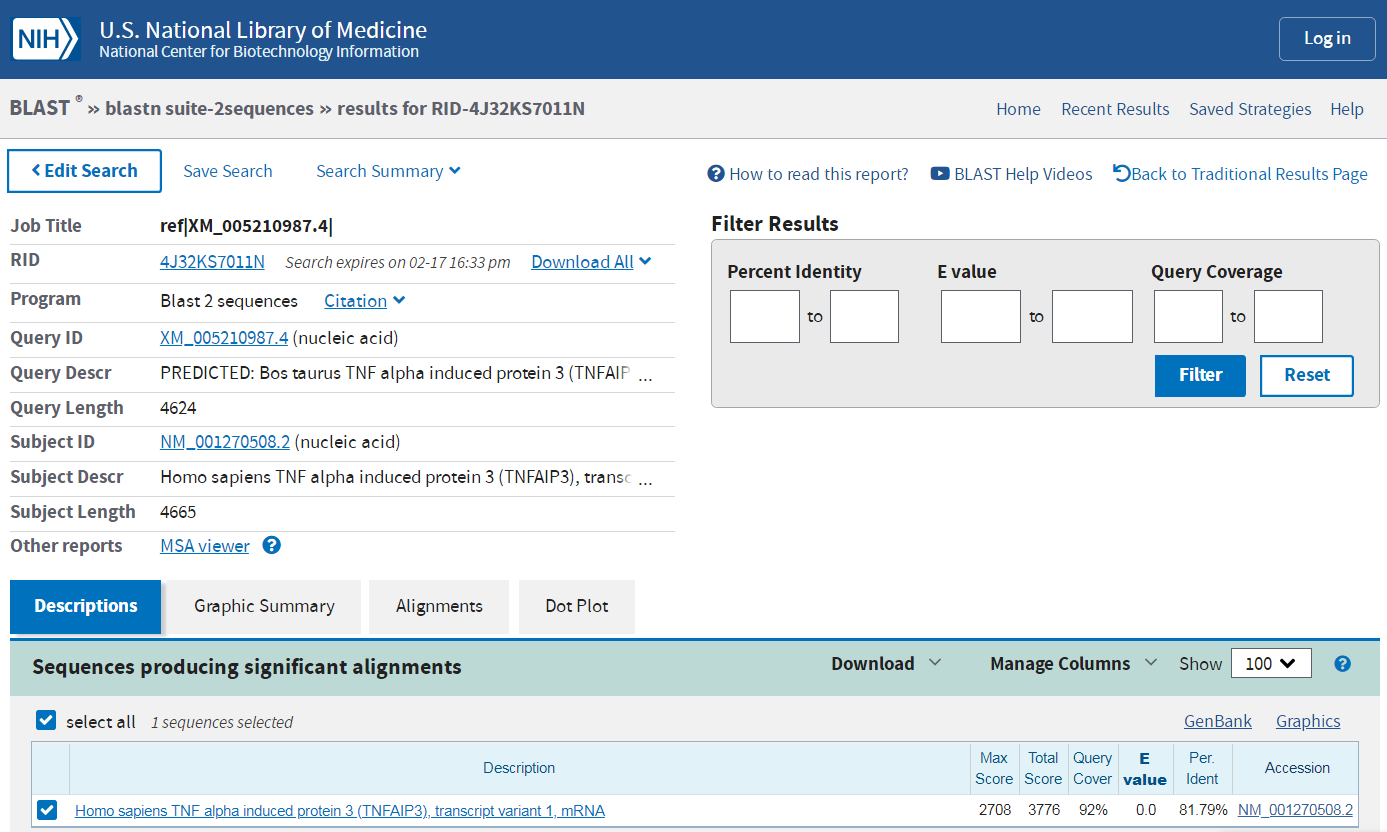

Supplement: Supplementary file 3 — Additional file 3:Homology alignment of miR-125b and TNFAIP3 gene in bovine and human.A Alignment of mature sequence of bovine miR-125b and human miR-125b. B Homology of complete TNFAIP3 mRNA in bovine and human. [file 13567_2021_992_MOESM3_ESM.docx]
