# Supplementary material for: MiR-125b regulates inflammation in bovine mammary epithelial cells by targeting the NKIRAS2 gene
Source: Vet Res. 2021 Sep 17;52:122. doi: 10.1186/s13567-021-00992-0 (PMC8447609; doi:10.1186/s13567-021-00992-0)
Supplement: Supplementary file 2 — Additional file 2: Protocol of total RNA isolation and RT-qPCR. [file 13567_2021_992_MOESM2_ESM.docx]

**Total RNA Isolation and RT-qPCR**

**Isolation of Total RNA**

Total RNA was extracted from mimic or inhibitor transfected bovine mammary epithelial cells (bMEC) using the Trizol reagent (Invitrogen, Carlsbad, CA, USA). The quality of RNA was assessed using a BioSpec-nano Micro-volume UV-Visible Spectrophotometer (Shimadzu Scientific Instruments, Shimadzu, Japan). For all samples, the OD_260/280_ ratio was between 1.8 and 2.0. The integrity of RNA in the samples was assessed by electrophoresis on 2% agarose gels. Each RNA sample was diluted with RNase-free ddH_2_O to 1000 ng/μL, and either used immediately or frozen at −80 °C for future use.

**gDNA Eraser and First-Strand cDNA synthesis**

First-Strand cDNA Synthesis was performed using the PrimeScript™ RT reagent Kit with gDNA Eraser (TaKaRa, Biotechnology Co. Ltd, Dalian, China) according to the manufacturer’s instructions. The details of the process are as follows:

***gDNA Eraser***

The gDNA Eraser reaction mixture (10 μL total volume contained 1.0 μL of total RNA (1 μg), 2.0 μL of 5 × gDNA Eraser Buffer, 1.0 μL of DNA Eraser, 7.0 μL of RNase Free H_2_O) was mixed with the total RNA extracted from the bMEC. The mixture was incubated at 42 °C for 2 min to promote the degradation of gDNA.

***First-Strand cDNA synthesis***

After gDNA Eraser reaction in the mixture completed, immediately following this step: 1.0 μL of PrimeScropt RT Enzyme Mix I (1.0 μL of 50.0 pmol RT Primer (Oligo dT and R6 mix for mRNA, stem-loop primer for miR-125b) mix, 4.0 μL of 5 × PrimeScript Buffer, and 4.0 μL of RNase Free H_2_O) was added to the 10 μL of gDNA Eraser reaction mixture mentioned above. Then, the mixture was incubated at 37 °C for 15 min, 85 °C for 5 s, then immediately placed on ice for 2 min. The cDNA was either used immediately or frozen at −20 °C.

**qPCR**

The cDNA was diluted 1:10 with DNase / RNase-free water. The qPCR was performed in an CFX96 Touch Real-Time PCR Detection System (BIO-RAD, USA) in a total volume of 20 μL (10 μL of 2 × SYBR Premix Ex TaqII (TliRNaseH Plus), 0.8 μL of PCR Forward Primer (10 μM), 0.8 μL of PCR Reverse Primer (10 μM), 2 μL of cDNA (100 ng/μL), and ddH_2_O to a final volume of 20 μL). The reaction mixtures were incubated at 95 °C for 30 s, followed by 40 cycles of 95 °C for 5 s, 60 °C for 34 s and 72 °C for 30 s. The primers used in this assay are listed in Additional file 4.

**qPCR Validation**

Quantitative PCR amplification efficiency (E) of each gene (or primer pair) in this study, including miRNA and mRNA, ranged from 93.7% to 104.90% with an R^2^ > 0.99. Both no-template control (NTC) and no-reverse transcriptase control (NRC) were included in all assay plates to verify the absence of reagent contamination or the generation of primer dimers. An amplification signal in the no template control was ignored as long as Cq values of NTC > 38, and the difference in Cq value between the NTC and the highest Cq > 6. At the end of each run, a dissociation melting curve of the product was determined. All melting curves showed a single peak, and were consistent with the presence of a single amplicon. All amplifications were run in triplicate, and any ambiguous curves were excluded.

**Data analysis**

All qPCR were performed in three independent experiments. The data are presented as fold differences relative to a reference gene based on the equation RQ = 2^−ΔΔCt^ [15]. The geometric mean of the two genes (*GADPH* and *UXT*), which have been previously validated as reference genes for qPCR of stimulated bMEC [2], was used to normalize the expression of mRNA. Negative amplification of a sample was assigned a Cq value of 40. Samples crossing the amplification detection threshold within 6 cycles of NTC amplification were excluded from the analysis.

Gene expression levels were evaluated in three replicates. All gene expression data are expressed as mean ± SD. GraphPad Prism 8.0 software (GraphPad Software, Inc., La Jolla, CA, USA) was used for statistical analysis and ordinary one-way ANOVA multiple comparisons were used to test the significance of differences. If the corrected *P* value was less than 0.05, the difference was deemed significant; if the corrected *P* value was less than 0.01, then the difference was deemed extremely significant.
