# Supplementary material for: MiR-125b regulates inflammation in bovine mammary epithelial cells by targeting the NKIRAS2 gene
Source: Vet Res. 2021 Sep 17;52:122. doi: 10.1186/s13567-021-00992-0 (PMC8447609; doi:10.1186/s13567-021-00992-0)
Supplement: Supplementary file 4 — Additional file 4: Alignment of 3´ UTR of bovine TNFAIP3 and human TNFAIP3. [file 13567_2021_992_MOESM4_ESM.docx]

Alignment of XM_005210987.4(bovine TNFAIP3) and NM_001270508.2(Human TNFAIP3)

Identity=69.45%(1287/1853) Gap=10.09%(208/2061)

-----------------

**3´UTR**

XM_005210987.4 TAAC........................................................

||||

NM_001270508.2 TAACCGGAAACAGGTGGGTCACCTCCTGCAAGAAGTGGGGCCTCGAGCTGTCAGTCATCA

Stop codon

XM_005210987.4 ............CCAACCCAGGGGGTCAACCCCAGACCCGGAGGGGCCTAGAGCCTTAGC

| | || | | | | | | || | |

NM_001270508.2 TGGTGCTATCCTCTGAACCCCTCAGCTGCCACTGCAACAGTGGGCTTAAGGGTGTCTGAG

XM_005210987.4 CCGTGGGGCGCCCCGGTGCCACTCCCGAGGGCCCTACCCTGCAGGCTGGG....CCTGGG

| | | | || || | | || |||| | || || ||

NM_001270508.2 CAGGAGAGGAAAGATAAGCTCTTCGTGGTGCCCACGATGCTCAGGTTTGGTAACCCGGGA

XM_005210987.4 CTGTCTGTGAGCCGGGGACGAAAGAAGCGCTCGGTCACCGGCCAGGAATCTGAGCAGAGC

||| | | ||||| | ||| | | || ||| ||| || ||||

NM_001270508.2 GTGTTCCCAGGTGGCCTTAGAAAGCAAAGCTTG.TAACTGGCAAGGGATGATGTCAGATT

XM_005210987.4 ..GTGTGACGGGCCGCGGGTTGCACAGGGTCAG...CCCGGAGCTGCTGCTCCTCCTCCC

| | | || | | || | | || ||| || ||

NM_001270508.2 CAGCCCAAGGTTCCTCCTCTCCTACCAAGCAGGAGGCCAGGAACTTCTTTGGACTTGGAA

XM_005210987.4 CTTCCGAGCAGAA.GGCTGGGAACCTCCAAGCCAGGACAGC.......CCCCACCATCTT

| | | || ||| | | || | || | || | | || | ||||

NM_001270508.2 GGTGTGCGGGGACTGGCCGAGGCCCCTGCACCCTGCGCATCAGGACTGCTTCATCGTCTT

XM_005210987.4 TAGAGA.AAAGAGAAAAGACGCAGGATCCGGCTGGACTGGGAAACCCAGAGCCAGTCC.C

|| |||| |||||||| || | || ||| ||| || ||||||||| || |

NM_001270508.2 GGCTGAGAAAGGGAAAAGACACACAAGTCGCGTGGGTTGGAGAAGCCAGAGCCATTCCAC

Predicted bta-miR-125b binding site 1#

XM_005210987.4 CTGCCCTCCCTCAGCATCTCTCGGAGACCAGGAGCTGAGGCCTCAGGGCCCGCAGGCCCT

|| ||||||| ||||||||||| |||| | ||| ||||| ||| |||||||

NM_001270508.2 CTCCCCTCCCCCAGCATCTCTCAGAGATGTGAAGCCAGATCCTCATGGCAGCGAGGCCCT

Predicted bta-miR-125b binding site 2#

XM_005210987.4 CTGCTGGAAACTCA.GGAAGCTCAGGGAGCACAGGCTCAGACACAGACTCAGAGACAGAC

|||| ||| |||| ||||||||||||| | || || | |||||||

NM_001270508.2 CTGCAAGAAGCTCAAGGAAGCTCAGGGAAAAT.GG.......ACGTATTCAGAGA.....

Identified has-miR-125b binding site (Haemmig et al., 2014, Kim et al., 2012)

XM_005210987.4 TCAGAGCATCAGTCAGTCGCGGTTTCGCCCTACTGGCCTCACTCCTCTCCCACGGACACG

| | || || ||||| |||||| ||| |||| ||| |||| ||

NM_001270508.2 .....GTGTTTGTAGTTCATGGTTTTTCCCTACCTGCCCGGTTCCTTTCCTGAGGACCCG

XM_005210987.4 GCAGAA..GCAGAACCATCCACAGACGGTGAT.........GCTGAGGCTGAGCACGATC

|||||| ||||||||||||| ||| ||||| |||||| |||| || | ||

NM_001270508.2 GCAGAAATGCAGAACCATCCATGGACTGTGATTCTGAGGCTGCTGAGACTGAACATGTTC

XM_005210987.4 ATAAAGAAAAGCAAACCAGCTGCTCTTTACAATATGCACCTTTCAAGAC.TCAGAACAGC

| | || | |||| |||||||||||| ||||||||||||| || | | |||| |

NM_001270508.2 ACATTGACAGAAAAACAAGCTGCTCTTTATAATATGCACCTTTTAAAAAATTAGAATATT

XM_005210987.4 .....GGGCAGATGTGTAACTCCT..GGTTACGGCTGTCTTCACTTCTAGAG...TTAAC

||| ||| ||||||||| | ||||| ||||||| ||||||| || ||| |

NM_001270508.2 TTACTGGGAAGACGTGTAACTCTTTGGGTTATTACTGTCTTTACTTCTAAAGAAGTTAGC

XM_005210987.4 TCGAACTGAGGTGTGCATACATTGTGTACATATACCACGGCCCCTTATATTCTGTATGAG

| ||||||||| || | | |||||||||||| | |||||| ||| ||||||||

NM_001270508.2 TTGAACTGAGGAGTA...AAAGTGTGTACATATATAATATACCCTTACATTATGTATGAG

XM_005210987.4 GGAATTTTTTTTTTTTTTTTTTGGTCATGTTGAGATGCTGCCCTAGAGGTTTTAAGTAAG

||| ||||||| | || |||| ||||||||||||| || | || |

NM_001270508.2 GGA...........TTTTTTTAAATTATATTGAAATGCTGCCCTAGAAGTAC..AATAGG

XM_005210987.4 AGGACTGACTAATAATAATAACCTATTTTCTGGTTGTTGTTGGGGCAGGAACTTCT.CAT

| | || | ||||||||||||| |||||||||||||||||||||| || ||| | ||

NM_001270508.2 AAGGCTAA...ATAATAATAACCTGTTTTCTGGTTGTTGTTGGGGCATGAGCTTGTGTAT

XM_005210987.4 AGACAGCTTGCGTGAACTCCACTGGCTGCTCTGTGGAATGGAAAT........TCCTGTG

| || |||||| | ||||| || ||||| | | || ||| | | ||||

NM_001270508.2 ACACTGCTTGCATAAACTCAACCAGCTGCCTTTTTAAAGGGAGCTCTAGTCCTTTTTGTG

XM_005210987.4 TAAATCACTTTATTTATTTTATTACAAACTTTAAGGTTATTTAAATGAAGATGTTTCTTC

||| ||||||||||||||||||||||||||| ||| |||||||| ||||||| |||||||

NM_001270508.2 TAATTCACTTTATTTATTTTATTACAAACTTCAAGATTATTTAAGTGAAGATATTTCTTC

XM_005210987.4 TGGTCTTCAGAAAATGCTTGCTGTAGTGTCCTCTTGAGATAAAATAAG.ACTGACTATCA

| ||| |||||| || ||||| ||| ||||| || || || |||

NM_001270508.2 AGCTCTGGGGAAAAT....GCCACAGTGTTCTCCTGAGAGAACATCCTTGCTTTGAGTCA

XM_005210987.4 GAA.GCAGGCAAGTTCCTGATAACGGTGGGGGAGTCACCTTTCTCCGTGCACTTTGCTTG

| | ||||||||||||| || | | | | | | | | | |||||| |

NM_001270508.2 GGCTGTGGGCAAGTTCCTGACCACAGGGAGTAAATTGGCCTCTTTGATACACTTTTGCTT

XM_005210987.4 CCTTCCCTAGGAAAGAAACAGTCTCTTCCACAGTAAATCTATTAAAATCTCACACTTTCT

| |||| ||||||||| | | | |||| ||| | || | || | | | |

NM_001270508.2 GCCTCCCCAGGAAAGAAGGAATTGCATCCAAGGTATACATAC..ATATTCATCGATGTTT

XM_005210987.4 CGTGAGACTTTTGGCTAGAGCCACTTCCCAGCCCTTCAGTGAGAAGCCATTCTCCACAGT

|||| || | | | ||||| | | | | || | || ||| |

NM_001270508.2 CGTGCTTCTCCTTATGAAA......CTCCAGCTATGTAATAAAAAACTATACTCTGTGTT

XM_005210987.4 GTATAAATGGTTCTGTGTATCCTACCTCCTCAGAGAGACGATTGGGAAGGAGCAGAGATG

| | |||| |||| || ||||||||||| |||| ||| |||||||||||| ||||

NM_001270508.2 CTGTTAATGCCTCTGAGTGTCCTACCTCCTTGGAGATGAGATAGGGAAGGAGCAGGGATG

XM_005210987.4 CGATCGGGGAAGGTCACGGGGAAAGATGTTGCCTTTGACGAAGGGTTTGTTTGCTGTGTT

|| || | |||||| ||||||||||| |||||| || || ||| ||| |||| |

NM_001270508.2 AGACTGGCAATGGTCACAGGGAAAGATGTGGCCTTTTGTGATGGTTTTATTTTCTGT..T

XM_005210987.4 GATGCTGCGTCCTGGGGTGCGGAGCGGTGACCTTGCAGCC......AGCTTAGCACTGGA

| ||| ||||||||| | | || |||| || | | | | | |||

NM_001270508.2 AACACTGTGTCCTGGGGGGGCTGGGAAGTCCCCTGCATCCCATGGTACCCTGGTATTGGG

XM_005210987.4 ACAGCACAACCTTGTAACCATGAGTATGAGGAAACCTCTGTCTGTCCGTGGCCCGCAGCT

|||||| || | ||||||||||||||||||||| |||| ||||| |||| ||| |

NM_001270508.2 ACAGCAAAAGCCAGTAACCATGAGTATGAGGAAATCTCTTTCTGTTGCTGGCTTACAGTT

XM_005210987.4 TCTCTGTGTGTTTTGCG.TTACTGTCATACTTGTGCTAG..GAAAAAAAAAAAAAGAAGG

|||||||||| |||| | || |||||||| ||| |||| ||||||||||||| || ||

NM_001270508.2 TCTCTGTGTGCTTTGTGGTTGCTGTCATATTTGCTCTAGAAGAAAAAAAAAAAAGGAGGG

XM_005210987.4 GAAGCACATTGTCCCCAGAGGTAAAGGCTGCTATTTTTTTGTTGTTGGTCTGAACTTATG

||| |||| ||||||||| |||||||||| ||||| | |||||| |||||||

NM_001270508.2 GAAATGCATTTTCCCCAGAGATAAAGGCTGCCATTTT......GGGGGTCTGTACTTATG

XM_005210987.4 ACCTGAAAATCTTG......CCTAATGCTCCATAGCCTTTACTGGTCTGACATGATGTGT

||||||||| || | ||| || || |||||||||| | | |

NM_001270508.2 GCCTGAAAATATTTGTGATCCATAACTCTACACAGCCTTTACTCATACTATTAGGCACAC

XM_005210987.4 TTTCCTCTTGAAAATCCATACACCTTTTCCCTTGGCATATCCCTTTA.TGTCTTTCTAAA

||||| ||| | || || |||| || | | ||| | || | |||||| |||

NM_001270508.2 TTTCCCCTTAGAGCCCCCTAAG..TTTTTCCCAGACGAATCTTTATAATTTCTTTCCAAA

XM_005210987.4 GATTTCAAATAAACGTCAGTGTTTTCATTTAGTTCTTTTAAAGTTTCTATTTTAATATTT

||| ||||||||| ||||||||||||| || |||| |||||||| ||| |||||||||

NM_001270508.2 GATACCAAATAAACTTCAGTGTTTTCATCTAATTCTCTTAAAGTTGATATCTTAATATTT

XM_005210987.4 TATGTGTATCAATATTTTCATTCTTAATGTGAATAAATGGAAT.ATTTATGCTTATTATA

| ||| |||| ||||| ||||||||||||||| ||| | ||| |||||| |||||||||

NM_001270508.2 TGTGTTGATCATTATTTCCATTCTTAATGTGAAAAAAAGTAATTATTTATACTTATTATA

XM_005210987.4 CAGAACATTTGAAATTTGCACATTTAATTGTCTCTAATAGAAAACCGTTGACTCTCCTCT

| | |||||||||||||||||||||||||| |||||||||| || || | || |

NM_001270508.2 AAAAGTATTTGAAATTTGCACATTTAATTGTCCCTAATAGAAAGCCAC...CTATTCTTT

XM_005210987.4 ATTGGCTTTCTTCAAGTTTCCCTAAATTTAAATGTAACTTTTCACAAAAGTCAAAATTAA

|||| ||||||||||||| |||||| |||||||||||||||||| |||||| |||||

NM_001270508.2 GTTGGATTTCTTCAAGTTTTTCTAAAT..AAATGTAACTTTTCACAAGAGTCAACATTAA

XM_005210987.4 AAAGTAAATTATTTAA.....

||| ||||||||||||

NM_001270508.2 AAAATAAATTATTTAAGAACA
